# The role of venues in structuring HIV, sexually transmitted infections, and risk networks among men who have sex with men

**DOI:** 10.1186/s12889-018-5140-3

**Published:** 2018-02-07

**Authors:** Lydia N. Drumright, Sharon S. Weir, Simon D. W. Frost

**Affiliations:** 1 Department of Medicine, University of Cambridge, Addenbrookes Hospital, Box 157, Level 5, Hills Road, Cambridge, CB2 0QQ UK; 2 University of North Carolina – Chapel Hill, Chapel Hill, NC USA; 3 Department of Veterinary Medicine, University of Cambridge, Cambridge, UK

**Keywords:** Venues, Propensity score weighting, Marginal structural models, Causal inference, Social networks, HIV, Sexually transmitted infections, Methamphetamine

## Abstract

**Background:**

Venues form part of the sampling frame for time-location sampling, an approach often used for HIV surveillance. While sampling location is often regarded as a nuisance factor, venues may play a central role in structuring risk networks. We investigated individual reports of risk behaviors and infections among men who have sex with men (MSM) attending different venues to examine structuring of HIV risk behaviors. However, teasing apart ‘risky people’ from ‘risky places’ is difficult, as individuals cannot be randomized to attend different venues. However, we can emulate this statistically using marginal structural models, which inversely weight individuals according to their estimated probability of attending the venue.

**Methods:**

We conducted a cross-sectional survey of 609 MSM patrons of 14 bars in San Diego, California, recruited using the Priorities for Local AIDS Control Efforts (PLACE) methodology, which consists of a multi-level identification and assessment of venues for HIV risk through population surveys.

**Results and discussion:**

Venues differed by many factors, including participants’ reported age, ethnicity, number of lifetime male partners, past sexually transmitted infection (STI), and HIV status. In multivariable marginal structural models, venues demonstrated structuring of HIV+ status, past STI, and methamphetamine use, independently of individual-level characteristics.

**Conclusions:**

Studies using time-location sampling should consider venue as an important covariate, and the use of marginal structural models may help to identify risky venues. This may assist in widespread, economically feasible and sustainable targeted surveillance and prevention. A more mechanistic understanding of how ‘risky venues’ emerge and structure risk is needed.

**Electronic supplementary material:**

The online version of this article (10.1186/s12889-018-5140-3) contains supplementary material, which is available to authorized users.

## Background

The structure of sexual networks plays a central role in the transmission dynamics of sexually transmitted infections (STIs) and HIV [1]. There is an increasing body of literature that supports the role of venues, such as bars or the Internet, in structuring sexual networks through modifying the probability of meeting partners with certain risk characteristics, and hence increasing STI transmission risk [2]. Venues also form part of the sampling frame for time-location sampling, an approach often used for surveillance of HIV/STIs [3], for example as part of the National HIV Behavioral Surveillance System employed by the Centers for Disease Control and Prevention in the U.S. [4].^.^ Additionally, venues may provide unique opportunities for prevention [5].

Despite the apparent importance of venues, surprisingly few studies have directly addressed the extent to which the study population may be structured by venue. It may also be difficult to separate ‘risky people’ from ‘risky places’, as venues are comprised of the people who attend them, and patrons of different venues may differ in multiple characteristics. The difficulty in teasing apart the effects of individual and environment is acknowledged in the literature on neighborhood effects on health [6]. In this vein, Diez Roux [7] has outlined research directions for understanding the multilevel determinants of health, including (1) examining contexts other than neighborhoods; (2) improving measurement of group-level constructs; (3) applying techniques more appropriate for causal inference from observational data; (4) analyzing data from “natural experiments” involving exogenous variations in contextual characteristics; (5) examining dependencies between groups (such as spatial dependencies) and allowing for reciprocal relations between individuals and contexts; and (6) contrasting multilevel statistical models and complex systems models in the study of multilevel effects.

Individuals in a personal social network may contribute to the risk of HIV/STI transmission, leading to the concept of a ‘risk network’ [8–10]. To address the importance of bars and clubs in structuring the HIV/STI risk networks of men who have sex with men (MSM), we conducted a Priorities for Local AIDS Control Efforts (PLACE) [5, 11] study in San Diego, California. Based on location identified by men in street interviews, we interviewed patrons in 14 venues reported as places where men could meet male sexual partners. We examined how patron characteristics were structured by venue in terms of demographics, risk behaviours, and self-reported HIV status and STI history using different modelling techniques. Even though the venues we considered were composed of bars and clubs, there was extensive heterogeneity in study participant characteristics across venues. We specifically focused on the correlation between venue and use by specific age groups, which introduced problems of collinearity in statistical models that considered both age and venue as predictor variables, and how this may be overcome through the use of causal inference approaches, as highlighted by Diez Roux [7].

## Methods

### Study design and population

Between October 2007 and March 2009, we used an enhanced version of the PLACE methodology [5, 11] to investigate the role of social networks and venues in structuring HIV risk networks among MSM. Briefly, PLACE consists of street-based cross-sectional interviews of the public to identify locations where people could go to meet new sexual partners, followed by interviews of staff and patrons in the identified meeting locations. As we were interested in MSM, we only included men in street and venue interviews, although any man agreeing to be interviewed was included, regardless of the sex of his sexual partners. Multiple venue types (bars, cafes, bathhouses, stores, parks, Internet websites, etc.) were identified by the street based interviews. Due to ethical concerns, logistic issues, social acceptability and/or the relatively low density of MSM in many of the named venues (e.g., Internet dating sites, bathhouses, parks, stores, cafes), we focused our patron interviews on bars and night clubs. In the street based interviews, 26 bars and clubs were identified, of which one had closed down. We obtained permission to visit 14 bars and night clubs, out of 16 of the most commonly identified, to interview staff and male patrons; the selection of venues was based on those most commonly identified by participants in the street-based interviews, and was limited to 14 due to time and budgetary constraints. The managers of two venues did not give their permission, on the basis that it may disturb their clientele. A given venue was visited on multiple occasions, in order to capture potential differences in clientele. The timing of these visits was informed through interviews with bar owners/managers as part of obtaining consent to interview participants in the venue. Between November 2007 and March 2009, 660 male patrons were interviewed in the 14 named venues. The method of study introduction to venue patrons (i.e. how patrons were approached and invited to participate) was tailored to the comfort of the venue managers or owners. In most venues interviewers were asked to enter the venue and distribute flyers or briefly introduce the study, in others interviewers were asked to distribute flyers/introduce the study at the door. The study was only introduced to male patrons, and any man who consented to participate was eligible. Participation rate was 98%, however due to intensive interest in the study, patrons more often approached interviewers than vice versa.

### Data collection

Venue patrons were interviewed by computer-assisted self-interview (CASI) using handheld computers (SNAP Software, Portsmouth, NH, USA) with a small team (4–5 individuals) of trained interviewers. Interviews were specifically developed for this study based on our previous HIV/STI research, information gathered in street interviews and in discussion with bar owners and managers. Questionnaires were the same in each venue, with the exception of the change in venue name (see questionnaire transcript in the Additional file 1). Patrons were queried by CASI about attendance at that and other venues in San Diego, their sexual behaviors in the past 12 months, meeting sexual partners within the venue in which we were interviewing and other venues, illicit substance use in the past 12 months in general and during sexual activity, STI history, and HIV status. All interviews were conducted anonymously, and were approximately 15 to 20 min in length. All participants were compensated US$5 for their time. The use of cash for compensation and the waiver of written consent was intended to increase anonymity of participants.

All participants in street interviews and who were patrons of bars completed verbal and documented anonymous (by a check box in the CASI) informed consent prior to participation. No record of participant name or signatures were collected, as this would reduce the anonymity of participation. Bar owners and/or managers who answered brief questions about the venue, theme nights, clientele, etc. and provided permission to interview in the venue, were asked to sign a paper informed consent for these activities, as they were not sharing personal information. Although the patron and street interviews were self-administered, a member of the research team was on hand to answer any questions. All questionnaires and recruitment activities were reviewed by community members to assess appropriateness and acceptability. The research protocol was approved by the University of California, San Diego Institutional Review Board, including the use of verbal consent only for venue patrons and men on the street. Further information on both the study population and the data collection have been described previously [12].

### Statistical analysis

Six hundred and sixty venue interviews were obtained. Of these, 609 participants reported having one or more male sexual partners in their lifetimes. Simple univariate comparisons by venue were examined using chi-squared tests (for categorical data), and Kruskal-Wallis tests (for continuous data). Tests for trend in venue characteristics by the median age of patrons were conducted using either a chi-squared test (for proportions) or by Kendall’s tau (for continuous data). There were a small amount of missing data (on age for two participants, on education for one participant, on the number of lifetime male partners for 16 participants, and on the number of male partners in the last 3 months for 52 participants), which was multiply imputed by Multivariate Imputation using Chained Equations (MICE) [13]. Pooling of parameter estimates and standard errors was performed using the method of Barnard and Rubin [14]. We analyzed three different outcomes; self-reported HIV status; history of any non-HIV STI; and methamphetamine use in general in the last 12 months, using logistic regression models. These models were either unweighted or were inverse probability weighted in order to control for heterogeneity in the model covariates across venues; the latter is sometimes known as a marginal structural model [15]. Inverse probability weights were calculated using a multinomial logistic model, with venue (*n* = 14) as the outcome, and age and college education as covariates; these covariates were selected on the basis of a backwards selection procedure. Analyses were performed using R [16], with the mice [17], ipw [18], and survey [19, 20] libraries.

## Results

### Description of venue types reported in street interviews

In October 2007, 296 men were interviewed on the street in order to identify different venue types where a man might go to meet a male sexual partner. Two hunderd-fifty three (85.5%) identified bars and night clubs, followed by the Internet (*n* = 99, 33.4%), coffee shops (67, 22.6%), parks (60, 20.3%), bathhouses (56, 18.9%), the street (38, 12.8%), the gym (34, 11.5%), adult book/video stores (29, 9.8%), grocery stores (28, 9.5%), the beach (18, 6.1%), private parties (13, 4.4%), public restrooms (13, 4.4%), work (13, 4.4%), sex clubs (4, 1.4%), telephone chatlines (4, 1.4%), and parking lots (2, 0.7%).

Twenty-six specific bars and night clubs were identified, which specifically catered to MSM and women who have sex with women, although women who have sex exclusively with men and men who exclusively have sex with women are frequently in attendance. They were located within specific communities in San Diego that are considered Lesbian Gay Bisexual and Transgender (LGBT) friendly. All of these venues were age restricted to 21 years and older, based on their alcohol and venue licensing classifications, but varied widely in other features. For example, some had regular theme nights; some served only bar food, while others had a full restaurant-style kitchen; and others had entertainment, such as ‘go-go’ or ‘pole’ dancers.

### Description of participants interviewed in bars and clubs

Six-hundred and nine participants, who reported at least one male sexual partner in their lifetime, were interviewed across 14 venues in San Diego between November 2007 and March 2009. Descriptive statistics for the sample are provided in Table 1. There was a great deal of variation in participants by age (median 31, interquartile range 25–42, range 19–78), with a high number of male sexual partners reported. 7.2% of participants reported having used methamphetamine in the past twelve months.

**Table 1:** Descriptive statistics of study participants (sample size, *n* = 609, except otherwise stated)

Variable	Median or value
Age (years) (*n* = 607)	31 (range 19–78)
Annual income (US$) (*n* = 597)	
< 10,000	23.6%
10,000–25,000	9.5%
25,000–50,000	28.1%
50,000–100,000	28.1%
More than 100,000	10.6%
Completed college (*n* = 608)	49.7%
White ethnicity	55.3%
Reported number of lifetime male sexual partners (*n* = 593)	30 (range 1–10,000)
Ever had sex with women (*n* = 593)	61.4%
Self-reported HIV+	12.6%
Self-reported past STI	34.6%
Used methamphetamine in last 12 months	7.2%
Met sexual partners at the venue	38.8%
Number of HIV+ individuals known (*n* = 606)	
0	24.5%
1	18.3%
2–5	38.9%
6–10	8.1%
11 or more	10.0%
Number of individuals known who have used methamphetamine (*n* = 603)	
0	58.7%
1	14.6%
2–5	19.1%
6–10	4.5%
11 or more	3.2%
Number of other venues (from 14) ever attended	
0	52.3%
1	5.9%
2–5	20.4%
6–10	17.2%
11–13	4.1%

### Description of venues by participant characteristics

**Fig. 1:**
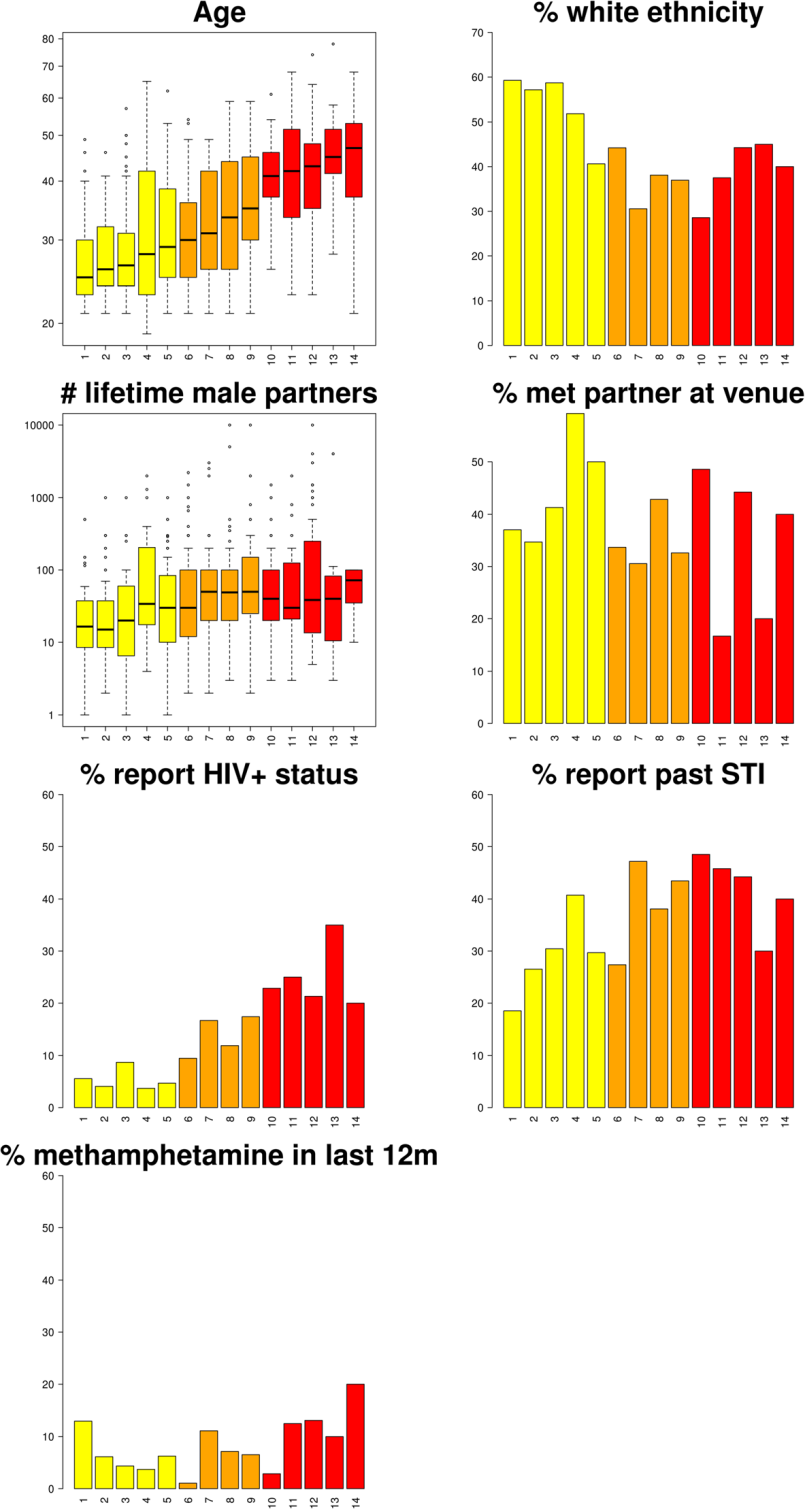
Participant characteristics across venues. The X-axis depicts the unique identifier number for each venue, ordered by median age which is consistent for all analyses. The Y-axis depicts the count or proportion. Bar colors depict the median age by venue (yellow, < 30; orange, 30–40; and red, > 40)

In simple univariate analyses, the 14 venues differed significantly by participants’ reported age, ethnicity, number of lifetime male partners, past STI infection, HIV+ status, number of HIV+ individuals known, and finding partners at that venue (Fig. 1). We classified each venue by the median age of the patrons interviewed there, and found that venues with older patrons were significantly more likely to have patrons of non-white ethnicity (*P* < 0.01), patrons who reported a greater number of lifetime male partners (*P* < 0.001), and patrons that reported HIV+ status or a history of STI (*P* < 0.001 for both). There was no significant trend by ‘venue age’ for either the proportion of participants meeting a partner at the venue (*P* = 0.42), or for methamphetamine use in general in the last twelve months (*P* = 0.21).

### Univariable models of HIV, STI, and methamphetamine use

In order to explore risk of HIV, STI, and methamphetamine use, we initially conducted univariable logistic regression analyses, with HIV+ status, past STI, or methamphetamine use in the last 12 months as the outcome, and venue, age, competed college (or not), 4 or more partners in the last 12 months, white ethnicity and whether individuals met partners at the venue as predictors in different models.

**Fig. 2:**
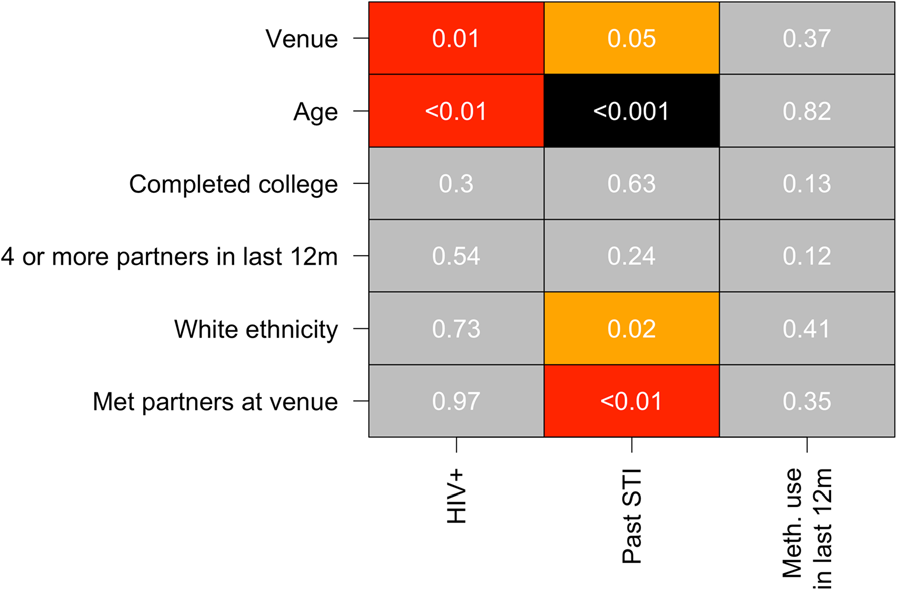
Statistical significance for univariate logistic regression analyses for: (a) HIV+ status; (b) past STI; and (c) methamphetamine use in the last 12 months as the outcome. Colors represent level of statistical significance (gray, *P* > 0.05; orange, 0.05 > *P* > 0.01; red, 0.01 > *P* ≥ 0.001; and black *P* < 0.001)

Venue and age were significantly associated with HIV+ status and past STI (Fig. 2). White ethnicity and meeting partners at the interview venue were also associated with a higher odds of past STI (odds ratio (OR) 1.36 and 1.35 respectively). There were no apparent associations (*P* > 0.05) with methamphetamine use. However, as shown in Fig. 1, venues varied dramatically by age, and therefore it was not clear from these univariable models whether associations with venue were being driven by differences in individual-level characteristics, or whether the venue was independently associated with risky behaviors.

### Multivariable models

A classical approach to deal with confounding is simply to include multiple predictor variables. As specific venues were of interest, we included venue as a categorical variable with 14 levels. We included variables significant at *P* < 0.1 in multivariable logistic regression analyses for HIV+ status and past STI. For HIV+ status, venue remained significant (*P* = 0.04), but age was not (*P* = 0.90). For past STI, while age remained statistically significant (OR = 1.3 per 10 years, *P* < 0.001), venue was not statistically significant (*P* = 0.47). Similar results were obtained with a generalized linear mixed model, in which venue was treated as a random effect, which has the effect of smoothing log-odds ratios for specific venues towards zero (results not shown).

However, collinearity between multiple variables based on our descriptive analyses led to the concern that logistic regression, which is known to give biased estimates of standard errors when collinearity is present, was providing potentially misleading results. Age and education (completed college or not) demonstrated significant associations with venue in a multinomial logistic regression model, suggesting that collinearity may present a problem with the interpretation of these standard models.

### Causal inference via marginal structural models

**Fig. 3:**
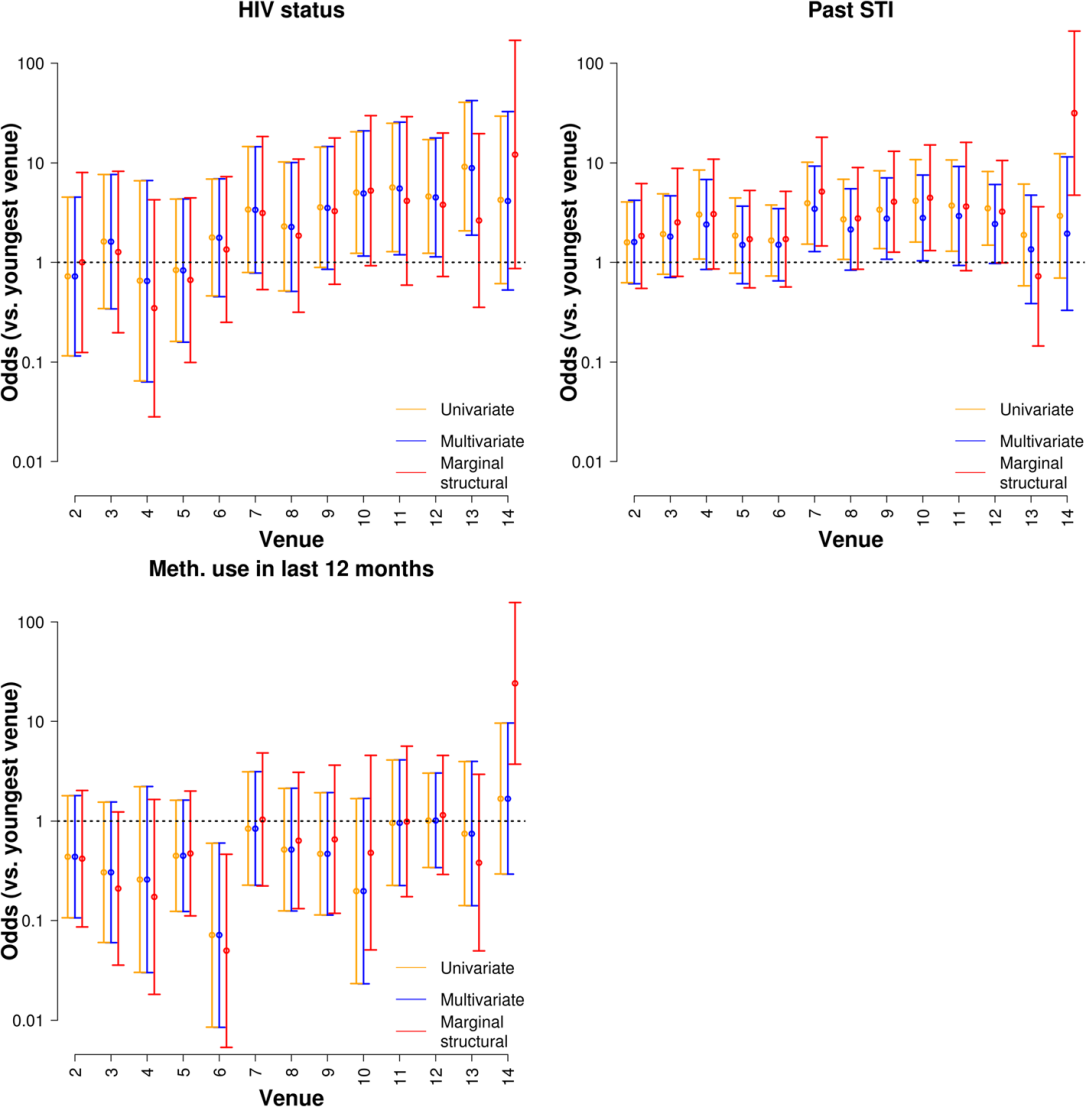
Odds ratios by venue (youngest venue (1) = reference) for HIV+ status, past STI, and use of methamphetamine in the past 12 months by statistical model. Estimates are shown for a univariate model (orange), a multivariate model (blue), and a marginal structural model (red). Whiskers indicate 95% confidence intervals

To determine whether venue was independently associated with self-reported HIV status, history of STI, and methamphetamine use in the last 12 months, we fitted a marginal structural model, which can be used as an alternative to multivariate regression models when collinearity in the predictors is a problem [15]. In this setting, the use of marginal structural models emulates randomizing individuals to venues, such that individuals in different venues are balanced in terms of confounding variables, and is more robust to colllinearity than standard fixed or random effects models. We used a multinomial logistic model with venue as the outcome, and age and education as predictors, to construct weights for a marginal structural model. This approach identified significant associations with venue, independently of age and education, for HIV+ status (*P* = 0.04), past STI (*P* < 0.01) as well as methamphetamine use (*P* < 0.001) (see Fig. 3, which also depicts 95% confidence intervals). Particularly dramatic effects were found for comparison between venue 1, the venue with the youngest median age of participants, and venue 14, with the oldest median age of participants. After controlling for age as a risk for HIV infection and education in a marginal structural model, venue 14 was associated with much higher odds of HIV+ status, past STI, and methamphetamine use compared to venue 1.

## Discussion

We have demonstrated that, in a time-location sample of MSM, venue location structured the patrons with respect to demographics, HIV/STI, and risk factors. This finding would have been masked by traditionally controlling for venue in a standard logistic regression analysis or missed had venue not been considered at all, which has important implications for studies that employ time-location sampling.

It is uncommon to examine time-location sampled data on a venue-by-venue basis, and the impact of venue-level heterogeneity on conclusions is rarely considered. This is particularly important when considering studies that attempt to estimate prevalence of HIV/STI, other diseases, or risk behaviors among specific population groups. The study results may be sensitive to the specific choice of venues selected (e.g., bars attended by MSM) for sampling, or to errors in the generation of the sampling frame. As information on the venue at which patrons are interviewed is normally collected, this should also be included in the statistical analysis, and approaches that are robust to collinearity, such as marginal structural models, used when appropriate. Karon [21, 22] proposed a weighting approach to correct for differential frequencies of venue attendance and clustering by venue, in order to generalize from a time-location sample to the population. Our focus is slightly different, in that we are primarily interested in those who attend venues, rather than the population as a whole, and the role of venues in structuring the venue-goers, rather than treating venue as a ‘nuisance factor’.

Our study also exemplified the problems involved in teasing apart the role of individuals from their venue using standard analytical techniques for epidemiological studies. As demographics, sexual history and disease status varied by venue, standard statistical models generated unstable parameter estimates when multiple variables were introduced into the model. The use of marginal structural models allowed us to tease apart venue from individual-level factors. Moreover, marginal structural models can easily be implemented using standard statistical software.

Even though venue was independently associated in statistical analyses, venue may simply be a proxy for individual-level variation that is not captured using the survey instrument. This is not to understate the importance of collecting information on interview venue; individuals may find it difficult or even impossible to describe their risk networks, which may be captured, albeit only approximately, by the venues that they attend.

Of particular interest is the association between venue and methamphetamine use. While the use of a marginal structural model had modest effects on the estimates of odds ratios for many of the venues compared to the use of a standard multivariable model with respect to STI history and HIV status, the odds ratio for methamphetamine use amongst patrons in venue 14 (venue with the oldest clientele on average) was dramatically higher in the marginal structural model. Thus, the approaches used here can be used to identify high-risk venues through surveillance using PLACE methodology and marginal structural models, which in turn could lead to prevention efforts tailored to specific venues.

While our study clearly demonstrates the importance of developing a better understanding of venues in HIV/STI risk, and methods to separate people from places, there are several limitations to our study. Data on HIV status and past STI were self-reported; individuals may believe they are HIV-infected when they are not, or they may be unaware of their HIV status. Additionally, while there were multiple venue types reported in our street interviews, we only interviewed patrons of 14 bars and clubs. This was due to types of venues that may not be appropriate for interview (e.g., bathhouses, public restrooms, parking lots, parks), and time and budgetary restrictions. It is also important to note that while we were given permission to interview in 14 venues out of 16 that we approached, one venue and the patrons were particularly cautious due to a previous and recent negative experience with a large HIV survey. However, participants frequently reported attending venues other than the interview venue (results not shown), giving us insights into venues not directly included in our sampling frame. With respect to behaviors such as methamphetamine use, which is often considered a ‘party drug’ in San Diego, it is unclear to what extent people choose to go to a venue due to others there who take methamphetamine (a type of social selection) versus the extent to which people change their behavior in response to others’ behaviors at the venues they attend (a form of social influence). As our data are cross-sectional rather than longitudinal, we have limited insights into the roles of social selection and social influence on methamphetamine use and other risk behaviors. The high participation rate in our study (98%) may also be driven by the familiarity that the local study population has with research studies, or the high level of support for HIV/STI research among MSM populations in San Diego, and hence may not be representative of all MSM. Similarly, while we endeavored to sample randomly within the venues, individuals often offered to participate, and hence our sample may not be strictly representative of a given venue.

In addition to physical venues, such as the bars and clubs considered in this study, or bathhouse, parks and other physical locations that could not practically be sampled, the Internet is becoming increasingly important as a ‘virtual’ venue to socialize and meet new sexual partners [23]. In an earlier study of recently HIV-infected MSM from San Diego [24], we showed that use of the Internet to find sexual partners was associated with more recent sexual partners, who were more likely to be HIV-negative; unpublished data from this study showed that most sexual partners were found on a small number of popular websites, and the population was not stratified by the website used; the Internet is likely to structure sexual networks in a different way, via partner selection criteria and individuals’ profiles on these websites.

While we have demonstrated structuring by venue, the question of how this structure arises remains. The structuring by venue is all the more remarkable given that individuals attend more than one venue. One could envisage how venues might attract patrons of a similar age to generate patterns similar to that seen in Fig. 1. Further research is needed to help understand the mechanistic basis underlying structuring of populations by venues. Longitudinal analysis of venues is likely to provide insight into whether patrons’ behaviors structure venue risk (i.e. social selection) or if venues structure individual patrons’ risk taking (i.e. social influence), and the balance between the two. Such studies would provide critical information in utilizing venues to stem the spread of HIV and STI infections.

Although social network characteristics have been highlighted as important in shaping HIV/STI risk [25], there are significant challenges to measure social networks, particularly in the context of infectious disease transmission [26]. The identification of HIV/STI risk structuring by venue – information that is easily collected – suggests that HIV/STI prevention activities could be delivered in local areas with defined venue structure. For example, campaigns targeting safer sex or methamphetamine cessation may reach more of the target population at venue 14, than venue 1. We suggest that rapid characterization of venue structuring can be conducted using PLACE methodology, which could be used to more effectively deliver interventions to target groups and thereby reduce the costs while potentially increasing the uptake of the intervention behavior.

## Conclusion

Venues, or sites that concentrate people, are important to both HIV/STI risk and prevention. In this study self-reported STI history, HIV+ status and use of methamphetamine in the past 12 months were significantly more likely in some venues than others after controlling for age and education in marginal structural models. More importantly, we know that demographics that are likely to be associated with HIV/STI risk, such as older age, are also likely to be associated with social venues, creating collinearity. Herein, we demonstrated appropriate statistical tools for such collinearity, and demonstrated how the use of naïve approaches can mask the importance in venue in structuring HIV/STI risk. It is critical that we understand how venues structure HV/STI transmission risk, so that we can harness these ‘spaces’ for HIV/STI prevention; therefore, we encourage robust and appropriate statistical modeling for time-location and other venue based sampling studies.

## Additional file

Additional file 1: Men’s Network Study Patron Interview Paper Transcript. This document is a transcript of the computer-assisted self-interview (CASI) administered to venue attendee participants in the Men’s Network Study. It provides the exact wording of the questions that participants would have been asked. The format and presentation, however, are quite different as it is not in CASI form. Skip patterns and venue names do not appear in this transcript. (PDF 725 kb)

## Abbreviations

CASI: Computer-assisted self-interview
HIV: Human immunodeficiency virus
HIV +: Human immunodeficiency virus positive (meaning infected with the human immunodeficiency virus)
MICE: Multiple imputation using chained equations
MSM: Men who have sex with men
OR: Odds ratio
STI: Sexually transmitted infection
