# Supplementary material for: The role of venues in structuring HIV, sexually transmitted infections, and risk networks among men who have sex with men
Source: BMC Public Health. 2018 Feb 7;18:225. doi: 10.1186/s12889-018-5140-3 (PMC5803997; doi:10.1186/s12889-018-5140-3)
Supplement: Additional file 1: — Men’s Network Study Patron Interview Paper Transcript. This document is a transcript of the computer-assisted self-interview (CASI) administered to venue attendee participants in the Men’s Network Study. It provides the exact wording of the questions that participants would have been asked. The format and presentation, however, are quite different as it is not in CASI form. Skip patterns and venue names do not appear in this transcript. (PDF 725 kb) [file 12889_2018_5140_MOESM1_ESM.pdf]

Supplemental Information: Men's Network Study Patron Interview Paper Transcript

From original CASI interview version 5

Published on 07-17-08

This is a transcript of the computer-assisted self-interview (CASI) for the Men's Network Study (MeNS) administered to participants in the venues where we interviewed. The skip patterns, presentation and structure of the interview are not fully represented in paper form. The below questions were in the complete set that were programmed into the CASI. Participants could decline to answer any questions except confirmation of participation. Please assume logical skip patterns based on responses to previous questions. Names of venues used were provided to us in confidential street interviews; participants were informed that names would not be published, therefore all name lists in the CASI have been removed on this paper form. In order to reduce the document length, instructions to participants have also been removed in most instances.

******** BEGIN INTERVIEWER SCREEN***********

I1 Enter Interviewer ID Number

I2 What is the name of this establishment?

Please make sure your spell the name correctly, it will be used in questions for the participants.

I3 Are you starting your first interview in this venue?

- Yes ☐
- No ☐

I4 Enter Today's Date as MM.DD.YYYY making sure that you place periods properly.

I5 Select the Day of the Week

- 1 Monday ☐
- 2 Tuesday ☐
- 3 Wednesday ☐
- 4 Thursday ☐
- 5 Friday ☐
- 6 Saturday ☐
- 7 Sunday ☐

I6 **Enter Time of Interview (24 hour clock HH:MM)**

I7 **Would you like to count the number of patrons in this venue or estimate percent full?**

Count (for 100 or fewer) ☐

Estimate % ☐

I8 **Please count the approximate number of patrons in this venue if 100 or fewer**.

____

I9 **How many men were attending the venue at this time?**

____

I10 **How many women were attending the venue at this time?**

____

I11 **Enter how full the venue was by percent maximum capacity.**

(Note you can go over %100 if you believe the venue exceeded its maximum capacity).

(Based on the names of venues entered we can provide the maximum capacity for the venue)

I12 **What percent of attendees were men?**

____

I13 **What percent of attendees were women?**

____

I14 **How many venue service people or attendants (e.g. bar tenders) were working?**

____

I15 **Was there anyone working at the door to check IDs (i.e., bouncer)**

Yes ☐

No ☐

I16 **Was there a theme or special event occurring in this venue at this time?**

Yes ☐

No ☐

I17 **Please enter the event or theme as it was presented in the venue.**

____

I18 **Did this person consent to volunteer?**

Yes ☐

No ☐

*************END INTERVIEWER SCREEN******************

S1 **Interviewer, please press the next button and then give the PDA to the interviewee.**

*********************BEGIN PARTICIPANT SCREENS***

S2 **Thank you for taking the time to speak with us and look at our questionnaire.**

This questionnaire is voluntary and will take approximately 10-15 minutes for you to complete.

Information from this questionnaire will help researchers at UCSD gain a better understanding of relationships between locations and sexual health in San Diego.

S3 **Your name or other personal identifiers will not be associated with this interview.**

Therefore this is considered an anonymous questionnaire.

To maintain your privacy, your interviewer will not be able to look back at your questionnaire. Your responses will be automatically coded as numbers in the computer.

No one will know your answers to these questions.

S4 You will be asked personal questions in this interview.

Remember, you can decline to answer any questions.

You will also be asked to provide the names of places where people may meet their partners.

This information is only for understanding where and how people meet their partners.

The places that you name will not be named in publications or presentations and no attempt will be made to change or interfere with specific places.

Q1 If you have any questions, please ask your interviewer and s/he will be happy to answer them.

If you would like to volunteer to complete this interview, please indicate that you are volunteering by marking the "yes" box below.

If you have changed your mind and do not want to complete this interview, please mark "no" below.

Yes	☐
No	☐

Q2 In what year were you born (YYYY)?

Q3 In what month were you born?

January	☐
February	☐
March	☐
April	☐
May	☐
June	☐
July	☐
August	☐
September	☐
October	☐
November	☐
December	☐
Decline	☐

Q4 **What is your ethnicity?**

- African American/ Black ☐
- Asian/ Pacific Islander ☐
- Caucasian/ White ☐
- Hispanic/ Latino ☐
- Native American/ Alaska Native/ American Indian ☐
- Other ☐

Q5 **What is the highest level of education that you have completed?**

- Less than high school ☐
- High school ☐
- Some college ☐
- Completed college ☐
- Some post-graduate work ☐
- Masters degree ☐
- Completed advanced degree (PhD, DVM etc.) ☐
- Decline ☐

Q6 **Are you currently working?**

- Full time ☐
- Parttime ☐
- Retired ☐
- On Disability ☐
- Not currently employed ☐
- Decline ☐

Q7 **Are you currently attending school?**

- Yes, high-school ☐
- Yes, college ☐
- Yes, post-high school training (other than college) ☐
- Yes, graduate school ☐
- No ☐

Q8 **What is your yearly income before taxes?**

Please do not include commas, dollar signs, or spaces.

____

Only report income that you have access to in your home.

If you are not sure of the exact amount, please estimate.

Q9 **Do you live in San Diego County?**

- Yes ☐
- No ☐
- Decline ☐

Q10 **What city and state (or country if outside of USA) do you currently live in?**

Q11 **Have you ever lived in San Diego County?**

- Yes ☐
- No ☐
- Decline ☐

S5 **In the next series of questions we are going to ask you about people you are acquainted with.**

When we refer to those who you are acquainted with, we mean people that you know by name and would stop and talk to for a moment if you ran into them on the street or in another social or public setting.

This includes friends, but NOT family. Please do not include family members as acquaintances.

Q12 **How many of the men that you are acquainted with are named Keith?**

- 0 ☐
- 1 ☐
- 2-5 ☐
- 6-10 ☐
- 11 or more ☐
- Decline ☐

Q13 **How many of the men that you are acquainted with are named Steve or Steven?**

0	☐
1	☐
2-5	☐
6-10	☐
11 or more	☐
Decline	☐

Q14 **How many of the men that you are acquainted with are named Kevin?**

0	☐
1	☐
2-5	☐
6-10	☐
11 or more	☐
Decline	☐

Q15 **How many of the men that you are acquainted with are named Mark, Marc or Marcus?**

0	☐
1	☐
2-5	☐
6-10	☐
11 or more	☐
Decline	☐

Q16 **How many of the women that you are acquainted with are named Susan, Suzy, Sue or Suzanne?**

0	☐
1	☐
2-5	☐
6-10	☐
11 or more	☐

Q17 **How many of the women that you are acquainted with are named Lisa?**

0	☐
1	☐
2-5	☐
6-10	☐
11 or more	☐
Decline	☐

Q18 **How many of the people that you are acquainted with are students?**

0	☐
1	☐
2-5	☐
6-10	☐
11 or more	☐
Decline	☐

Q17 **How many of the people that you are acquainted with have received an MD, PhD, or DVM?**

0	☐
1	☐
2-5	☐
6-10	☐
11 or more	☐
Decline	☐

Q18 **How many of the people that you are acquainted with are currently serving in the armed forces (military) either on active duty or in the reserves?**

0	☐
1	☐
2-5	☐
6-10	☐
11 or more	☐

Q19 **How many of the people that you are acquainted with are currently unemployed?**

0	☐
1	☐
2-5	☐
6-10	☐
11 or more	☐
Decline	☐

Q20 **How many of the people that you are acquainted with are Black or African American?**

0	☐
1	☐
2-5	☐
6-10	☐
11 or more	☐
Decline	☐

Q21 **How many of the people that you are acquainted with are Asian, Asian American or Pacific Islander?**

0	☐
1	☐
2-5	☐
6-10	☐
11 or more	☐
Decline	☐

Q22 **How many of the people that you are acquainted with are Hispanic or Latino?**

0	☐
1	☐
2-5	☐
6-10	☐
11 or more	☐

Q23 **How many of the people that you are acquainted with are White or Caucasian?**

0	☐
1	☐
2-5	☐
6-10	☐
11 or more	☐
Decline	☐

Q24 **How many of the people who you are acquainted with have been in prison or jail in the last 12 months?**

0	☐
1	☐
2-5	☐
6-10	☐
11 or more	☐
Decline	☐

Q25 **How many of the people that you are acquainted with have told you that they are HIV-positive?**

0	☐
1	☐
2-5	☐
6-10	☐
11 or more	☐
Decline	☐

Q26 **How many of the people that you are acquainted with have used marijuana (pot) on at least two different occasions in the past 12 months?**

0	☐
1	☐
2-5	☐
6-10	☐
11 or more	☐

Q27 **How many of the people that you are acquainted with have used poppers (nitrites) on at least two different occasions in the past 12 months?**

0	☐
1	☐
2-5	☐
6-10	☐
11 or more	☐
Decline	☐

Q28 **How many of the people that you are acquainted with have used methamphetamine (crystal, speed, meth) on at least two different occasions in the past 12 months?**

0	☐
1	☐
2-5	☐
6-10	☐
11 or more	☐
Decline	☐

S6 **In the next series of questions we will ask you about your recent and past sexual activity.**

When we refer to "sex" we are asking about the following activities:

Oral sex - stimulation of genitals with the mouth

Vaginal sex - insertion of a penis into a vagina

Anal sex - insertion of a penis into an anus

Q29 **Have you ever had sex in your lifetime?**

Yes	☐
No	☐
Decline	☐

Q30 **If you were to have sex, who would you choose to have it with?**

- Men only ☐
- Women only ☐
- Both men and women ☐
- I'm not sure ☐
- Decline ☐

Q31 **How many men have you had sex with in your lifetime?**

If you are not sure of the number, please estimate.

Q31 **How many women have you had sex with in your lifetime?**

If you are not sure of the number, please estimate.

Q32 **Who have you had sex with in the previous 12 months?**

- Men Only ☐
- Women only ☐
- Both men and women ☐
- Haven't had any sex in the past 12 months ☐
- Decline ☐

Q33 **How many different men have you had sex (oral or anal) with in each of the following time periods?**

	**Number of Men**
In the last 12 months	_______
Last 6 months	_______
Last 3 months	_______
Last 4 weeks	_______

Type the number, then press next. If 10,000 or more, type 9999. To decline to answer, type -1.

Q34 **In the past 12 months, that is from {v6month} until today, how many different men have you had the following types of sexual activity with? If you don't know, please guess.**

	**Number of Men**
Gave oral sex without a condom	_______
Gave oral sex using a condom	_______
Received oral sex without a condom	_______
Received oral sex using a condom	_______
Gave anal sex using a condom (top)	_______
Gave anal sex without a condom (top)	_______
Received anal sex using a condom (bottom)	_______
Received anal sex without a condom (bottom)	_______

Q35 **In the past 3 months, that is from {v3month} until today, how many different men have you had the following types of sexual activity with? If you don't know, please guess.**

	**Number of Men**
Gave oral sex without a condom	_______
Gave oral sex using a condom	_______
Received oral sex without a condom	_______
Received oral sex using a condom	_______
Gave anal sex using a condom (top)	_______
Gave anal sex without a condom (top)	_______
Received anal sex using a condom (bottom)	_______
Received anal sex without a condom (bottom)	_______

Enter a number for each category, the press next. To decline to answer type -1.

Q36 **Including all of the men that you had sexual contact with in the past 3 months, how many times in total did you have each of the following sexual activities, that is from {v3month} until today?** If you do not know, please guess.

	Number of Times
Gave oral sex without a condom	_______
Gave oral sex using a condom	_______
Received oral sex without a condom	_______
Received oral sex using a condom	_______
Gave anal sex using a condom (top)	_______
Gave anal sex without a condom (top)	_______
Received anal sex using a condom (bottom)	_______
Received anal sex without a condom (bottom)	_______

Q37 **How many different women have you had sex (oral, vaginal, or anal) with in each of the following time periods?**

	Number of Women
In the last 12 months	_______
Last 6 months	_______
Last 3 months	_______
Last 4 weeks	_______

Q38 **In the past 12 months, that is from {v6month} until today, how many different women have you had the following types of sexual activity with?**

	Number of Women
Gave oral sex without a barrier	_______
Gave oral sex using a barrier	_______
Received oral sex, no condom	_______
Received oral sex with condom	_______
Vaginal sex using a condom	_______
Vaginal sex without a condom	_______
Anal sex using a condom	_______
Anal sex without a condom	_______

Q39 **In the past 3 months, that is from {v3month} until today, how many different women have you had the following types of sexual activity with?**

	**Number of women**
Gave oral sex without a barrier	_______
Gave oral sex using a condom or barrier	_______
Received oral sex without a condom	_______
Received oral sex using a condom	_______
Vaginal sex using a condom	_______
Vaginal sex without a condom	_______
Anal sex using a condom	_______
Anal sex without a condom	_______

Type the number, then press next. If 10,000 or more, type 9999. To decline to answer, type -1.

Q40 **Including all of the women that you had sexual contact with in the last 3 months, how many times in total have you had each sexual activity from {v3month} until today?** If you do not know, please guess.

	**Number of Times**
Gave oral sex without a condom	_______
Gave oral sex using a condom	_______
Received oral sex without a condom	_______
Received oral sex using a condom	_______
Vaginal sex using a condom	_______
Vaginal sex without a condom	_______
Gave anal sex using a condom (top)	_______
Gave anal sex without a condom (top)	_______
Received anal sex using a condom (bottom)	_______
Received anal sex without a condom (bottom)	_______

Type the number, then press next. If 10,000 or more, type 9999. To decline to answer, type -1.

Q41 **Have you ever sold or traded sex for money, drugs, a place to stay, or something else?**

- Yes ☐
- No ☐

Q42 **Have you ever bought sex with money, drugs, or some other service or commodity?**

- Yes ☐
- No ☐
- Decline ☐

Q43 **Do you currently have a main partner (e.g., spouse, boyfriend, girlfriend, longterm partner)?**

- Yes ☐
- No ☐
- Decline ☐

Q44 **Is your main partner a man or woman?**

- Man ☐
- Woman ☐
- Transgender Man to Woman ☐
- Transgender Woman to Man ☐
- Decline ☐

Q45 **How old is your main partner?**

___

Q46 **What is your main partner's ethnicity?**

- African American/ Black ☐
- Asian/ Pacific Islander ☐
- Caucasian/ White ☐
- Hispanic/ Latino ☐
- Native American/ Alaska Native/ American Indian ☐
- Other ☐
- Decline ☐

Select all that apply, then press NEXT.

Q47 **How long have you and your main partner been together?**

Please answer in only one box, leaving the others blank.

Days ___

Weeks ___

Months ___

Years ___

Type the time into one time frame box only, then press NEXT.

To decline, type -1 in any one box.

Q48 **Where did you and your main partner meet?**

- adult book/ video store ☐
- bar or night club ☐
- bathhouse ☐
- beach ☐
- circuit party ☐
- coffee shop ☐
- community club, organization, or social function ☐
- through friends ☐
- grocery store ☐
- gym ☐
- Internet ☐
- park ☐
- parking lot ☐
- private party ☐
- public rest room ☐
- street ☐
- store other than grocery ☐
- telephone chatline ☐
- work or school ☐
- other ☐

Q49 **Did you meet your main partner in San Diego?**

Yes ☐

No ☐

Decline ☐

Q50 **In which adult store did you met your main partner?**

Name list removed for confidentiality ☐

Q51 **In which bar did you meet your main partner?**

Name list removed for confidentiality ☐

Q52 **In which bathhouse did you meet your main partner?**

Name list removed for confidentiality ☐

Q53 **At what beach did you meet your main partner?**

Name list removed for confidentiality ☐

Q54 **At which circuit party did you meet your main partner?**

____

Q55 **In which coffee shop did you meet your main partner?**

Name list removed for confidentiality ☐

Q56 **At which community club or organization did you meet your main partner**

Name list removed for confidentiality ☐

Q57 **In which grocery store did you meet your main partner?**

Name list removed for confidentiality ☐

Q58 **In which gym did you meet your main partner?**

Name list removed for confidentiality ☐

Q59 **On which Internet site did you meet your main partner?**

Name list removed for confidentiality ☐

Q60 **In which park did you meet your main partner?**

Name list removed for confidentiality ☐

Q61 **In which parking lot did you meet your main partner?**

Use cross-streets or store name and location

Q62 **In which public restroom did you meet your main partner?**

Use business names (e.g., San Diego Airport)

Q63 **Where on the street did you meet your main partner?**

Use cross-streets or a street and business location to describe where you met this partner.

Q64 **On which telephone chatline did you meet your main partner?**

Q65 **Which other place did you meet your main partner?**

Q66 **In which city and state (or country if outside of the USA) did you meet your main partner?**

Q67 **What type of sexual agreements (spoken or understood) regarding other people do you and your partner have?**

- We only have sex with each other ☐
- We can have sex with other people, but only together ☐
- We can have oral sex with other people, but no other types ☐
- We can have sex with other people, but only if we use a condom ☐
- We can have any type of sex with other people ☐
- We have no agreements ☐
- Other ☐

Q68 **Do you and your partner have the same HIV status or different (discordant) HIV statuses?**

- We have different HIV status (discordant) ☐
- We have the same HIV status (concordant) ☐
- I'm not sure, I don't know either of our statuses ☐
- I'm not sure, I don't know my HIV status ☐
- I'm not sure, I don't know my partner's HIV status ☐

Q69 **How long have you and your main partner been HIV sero-discordant (had different HIV statuses)?**

Please answer in only one box.

Days ___

Weeks ___

Months ___

Years ___

Q70 **What do you and your partner do to prevent HIV transmission?**

Not all of the answers may apply to you and your partner, remember these questions are for all partner types.

- We use condoms for all sex acts ☐
- We use condoms for all sex acts, except oral sex ☐
- We use condoms if the HIV positive person is the top in oral or anal sex ☐
- We use condoms if the HIV positive person is the top in anal sex ☐
- The HIV positive person is never the top in oral or anal sex ☐
- The HIV positive person is never the top in anal sex ☐
- We only have oral sex ☐
- The HIV positive person does note ejaculate (cum) inside the other person ☐
- We do not do anything to prevent transmission ☐
- The HIV positive person is taking HIV medication ☐
- Other ☐
- Decline ☐

Q71 How happy are you in your partnership with your main partner?

- Extremely happy ☐
- Very happy ☐
- Happy ☐
- Mostly happy ☐
- A little unhappy ☐
- Unhappy ☐
- Very unhappy ☐
- Extremely unhappy ☐
- Decline ☐

S7 In the next series of questions we will ask you about places that you may have gone to and if you met any new sex partners in these places.

Remember, these places will not be affected by being named in this questionnaire, nor will they be shared with anyone outside of the research team.

Q72 In the previous 12 months, which of the following types of "places" have you visited?

- adult book/ video stores ☐
- bars/night clubs ☐
- bathhouses/ sex clubs ☐
- beach ☐
- circuit / public parties ☐
- coffee shops ☐
- community clubs, organizations, or social functions ☐
- grocery stores ☐
- gym ☐
- Internet ☐
- parks ☐
- private parties ☐
- stores: home improvement, furniture, etc. ☐
- telephone chatlines ☐
- other ☐

Q73 **Were any of the places that you selected in the previous question in San Diego?**

- Yes ☐
- No ☐
- Decline ☐

Q74 **In the previous 12 months, where have you met a new sex partner?**

By "sex partner" we mean anyone you have had sex with, including a main partner, someone (man or woman) you dated, or someone you had a onetime sexual encounter with.

Please select all that apply, then press ENTER.

- adult book/ video stores ☐
- bars/night clubs ☐
- bathhouses/ sex clubs ☐
- beach ☐
- circuit parties ☐
- coffee shops ☐
- community clubs, organizations, or social functions ☐
- through friends ☐
- grocery stores ☐
- gym ☐
- Internet ☐
- parks ☐
- parking lots ☐
- private parties ☐
- public rest rooms ☐
- street ☐
- stores: home improvement, furniture, etc. ☐
- telephone chatlines ☐
- work ☐
- other ☐
- Decline ☐
- Met no new partners in the previous 12 months ☐

Q75 **There are many reasons that someone may not meet a new sexual partner in a 12 month time period in the places mentioned above.**

For what reason(s) do you think that you have not met a new sex partner in the last 12 months?

- I have met a new partner in the last 12 months, just not in those places ☐
- I'm in a longterm partnership ☐
- I was not interested in having a partner in the last 12 months ☐
- I was not ready to have a partner in the last 12 months ☐
- I would like to have a partner, but didn't meet the right person in the last 12 month ☐
- I tried, but could find anyone who was willing to have sex with me in the last 12 months ☐
- Other ☐
- Decline ☐

Q76 **Please indicate which adult stores you have visited in the previous 12 months and which you have met a new sex partner at in the previous 12 months.**

	Visited	Met New Sex Partner
Name list removed for confidentiality	☐	☐

In the "visited" column, mark all the places visited in the past 12 months.

In the "met new sex partner" column, mark all places where you met someone new in the past 12 months, then press NEXT.

If you did not meet any partners in the last 12 months in an adult store, please mark "met no partners".

Q77 **Please mark which bars you have visited in the previous 12 months and which you have met a new sex partner at in the previous 12 months.**

	Visited	Met New Sex Partner
Name list removed for confidentiality	☐	☐

In the "visited" column, mark all the places visited in the past 12 months.

In the "met new sex partner" column, mark all places where you met someone new in the past 12 months, then press NEXT.

If you did not meet any partners in the last 12 months in a bar/night club, please mark "met no partners".

Q78 **Please indicate which bathhouse(s) you have visited in the previous 12 months and which you have met a new sex partner at in the previous 12 months.**

	visited	Met New Sex Partner
Name list removed for confidentiality	☐	☐

In the "visited" column, mark all the places visited in the past 12 months.

In the "met new sex partner" column, mark all places where you met someone new in the past 12 months, then press NEXT.

Q79 **Please indicate which beach(es) you have visited in the previous 12 months and which you have met a new sex partner at in the previous 12 months.**

	Visited	Met New Sex Partner
Name list removed for confidentiality	☐	☐

In the "visited" column, mark all the places visited in the past 12 months.

In the "met new sex partner" column, mark all places where you met someone new in the past 12 months, then press NEXT.

If you did not meet any partners in the last 12 months at a beach, please mark "met no partners".

Q80 **Please indicate which circuit/ public party(ies) you have visited in the previous 12 months and which you have met a new sex partner at in the previous 12 months.**

	Visited	Met New Sex Partner
Name list removed for confidentiality	☐	☐

In the "visited" column, mark all the places visited in the past 12 months.

In the "met new sex partner" column, mark all places where you met someone new in the past 12 months, then press NEXT.

Q81 **Please indicate which cafe/ coffee shop(s) you have visited in the previous 12 months and which you have met a new sex partner at in the previous 12 months.**

	Visited	Met New Sex Partner
Name list removed for confidentiality	☐	☐

In the "visited" column, mark all the places visited in the past 12 months.

In the "met new sex partner" column, mark all places where you met someone new in the past 12 months, then press NEXT.

If you did not meet any partners in the last 12 months at a cafe, please mark "met no partners".

Q82 **Please indicate which community clubs/organizations you have visited in the previous 12 months and which you have met a new sex partner at in the previous 12 months.**

	Visited	Met New Sex Partner
Name list removed for confidentiality	☐	☐

In the "visited" column, mark all the places visited in the past 12 months.

In the "met new sex partner" column, mark all places where you met someone new in the past 12 months, then press NEXT.

If you did not meet any partners in the last 12 months at a community club/organization, please mark "met no partners".

Q83 **Please indicate which grocery stores you have visited in the previous 12 months and which you have met a new sex partner at in the previous 12 months.**

	Visited	Met New Sex Partner
Name list removed for confidentiality	☐	☐

In the "visited" column, mark all the places visited in the past 12 months.

In the "met new sex partner" column, mark all places where you met someone new in the past 12 months, then press NEXT.

If you did not meet any partners in the last 12 months at a grocery store, please mark "met no partners".

Q84 **Please indicate which gyms you have visited in the previous 12 months and which you have met a new sex partner at in the previous 12 months.**

	Visited	Met New Sex Partner
Name list removed for confidentiality	☐	☐

In the "visited" column, mark all the places visited in the past 12 months.

In the "met new sex partner" column, mark all places where you met someone new in the past 12 months, then press NEXT.

If you did not meet any partners in the last 12 months at a gym, please mark "met no partners".

Q85 **Please indicate which Internet sites you have visited in the previous 12 months and which you have met a new sex partner at in the previous 12 months.**

	Visited	Met New Sex Partner
Name list removed for confidentiality	☐	☐

In the "visited" column, mark all the places visited in the past 12 months.

In the "met new sex partner" column, mark all places where you met someone new in the past 12 months, then press NEXT.

If you did not meet any partners in the last 12 months on the Internet, please mark "met no partners".

Q86 **Please indicate which parks you have visited in the previous 12 months and which you have met a new sex partner at in the previous 12 months.**

	Visited	Met New Sex Partner
Name list removed for confidentiality	☐	☐

In the "visited" column, mark all the places visited in the past 12 months.

In the "met new sex partner" column, mark all places where you met someone new in the past 12 months, then press NEXT.

Q87 **Please indicate which parking lots you have met a new sex partner in in the previous 12 months. Please specify business and location or business and cross streets (e.g., Central & 5th)**

To decline, type "decline".

If outside San Diego County, type the name of the city**.**

Q88 **Please indicate which types of private parties have been to in the previous 12 months and which you have met a new sex partner at in the previous 12 months.**

	Visited	Met New Sex Partner
My party	☐	☐
Friends parties	☐	☐
Friend of a friends parties	☐	☐
Parties advertised on the Internet	☐	☐
Parties advertised in a paper or flyer	☐	☐
Other	☐	☐
Met no partners at a party last 12 months	☐	☐
Decline	☐	☐

In the "visited" column, mark all the places visited in the past 12 months.

In the "met new sex partner" column, mark all places where you met someone new in the past 12 months, then press NEXT.

Q89 **Please indicate which public rest rooms you have met a new sex partner at in the previous 12 months.**

Please indicate the business and location of the rest room.

To decline, type "decline".

____

Q90 **Please indicate which street locations you have met a new sex partner at in the previous 12 months.**

Please indicate the cross-streets or street and nearby landmark/business.

If outside of San Diego County, type the name of the city.

____

Q91 **Please indicate which telephone chatlines you have visited in the previous 12 months and which you have met a new sex partner at in the previous 12 months.**

____

Q92 **Please indicate which adult stores you have visited in the previous 12 months and which you have looked for a new sex partner at in the previous 12 months.**

	Visited	Looked for New Sex Partner
Name list removed for confidentiality	☐	☐

In the "visited" column, mark all the places visited in the past 12 months.

In the "looked for new sex partner" column, mark all places where you looked for someone new in the past 12 months, then press NEXT.

Q93 **Please mark which bars you have visited in the previous 12 months and which you have looked for a new sex partner at in the previous 12 months.**

	Visited	Looked for New Sex Partner
Name list removed for confidentiality	☐	☐

In the "visited" column, mark all the places visited in the past 12 months.

In the "looked for new sex partner" column, mark all places where you met someone new in the past 12 months, then press NEXT.

Q94 **Please indicate which bathhouse(s) you have visited in the previous 12 months and which you have looked for a new sex partner at in the previous 12 months.**

	visited	Looked for New Sex Partner
Name list removed for confidentiality	☐	☐

In the "visited" column, mark all the places visited in the past 12 months.

In the "met new sex partner" column, mark all places where you met someone new in the past 12 months, then press NEXT.

Q95 **Please indicate which beach(es) you have visited in the previous 12 months and which you have looked for a new sex partner at in the previous 12 months.**

	Visited	Looked for Partner
Name list removed for confidentiality	☐	☐

In the "visited" column, mark all the places visited in the past 12 months.

In the "looked for new sex partner" column, mark all places where you looked for someone new in the past 12 months, then press NEXT.

Q96 **Please indicate which circuit/ public party(ies) you have visited in the previous 12 months and which you have looked for a new sex partner at in the previous 12 months.**

	Visited	Looked for New Sex Partner
Name list removed for confidentiality	☐	☐

In the "visited" column, mark all the places visited in the past 12 months.

In the "looked for new sex partner" column, mark all places where you looked for someone new in the past 12 months, then press NEXT.

Q97 **Please indicate which cafe/ coffee shop(s) you have visited in the previous 12 months and which you have looked for a new sex partner at in the previous 12 months.**

	Visited	Looked for New Sex Partner
Name list removed for confidentiality	☐	☐

In the "visited" column, mark all the places visited in the past 12 months.

In the "looked for new sex partner" column, mark all places where you looked for someone new in the past 12 months, then press NEXT.

Q98 **Please indicate which community clubs/organizations you have visited in the previous 12 months and which you have looked for a new sex partner at in the previous 12 months.**

	Visited	Looked for New Sex Partner
Name list removed for confidentiality	☐	☐

In the "visited" column, mark all the places visited in the past 12 months.

In the "looked for new sex partner" column, mark all places where you looked for someone new in the past 12 months, then press NEXT.

Q99 **Please indicate which grocery stores you have visited in the previous 12 months and which you have looked for a new sex partner at in the previous 12 months.**

	Visited	Looked for New Sex Partner
Name list removed for confidentiality	☐	☐

In the "visited" column, mark all the places visited in the past 12 months.

In the "looked for new sex partner" column, mark all places where you looked for someone new in the past 12 months, then press NEXT.

Q100 **Please indicate which gyms you have visited in the previous 12 months and which you have looked for a new sex partner at in the previous 12 months.**

	Visited	Looked for New Sex Partner
Name list removed for confidentiality	☐	☐

In the "visited" column, mark all the places visited in the past 12 months.

In the "looked for new sex partner" column, mark all places where you looked for someone new in the past 12 months, then press NEXT.

Q101 **Please indicate which Internet sites you have visited in the previous 12 months and which you have looked for a new sex partner at in the previous 12 months.**

	Visited	Looked for New Sex Partner
Name list removed for confidentiality	☐	☐

In the "visited" column, mark all the places visited in the past 12 months.

In the "looked for new sex partner" column, mark all places where you looked for someone new in the past 12 months, then press NEXT.

Q102 **Please indicate which parks you have visited in the previous 12 months and which you have looked for a new sex partner at in the previous 12 months.**

	Visited	Looked for New Sex Partner
Name list removed for confidentiality	☐	☐

In the "visited" column, mark all the places visited in the past 12 months.

In the "looked for new sex partner" column, mark all places where you looked for someone new in the past 12 months, then press NEXT.

Q103 **Please indicate which types of private parties have been to in the previous 12 months and which you have looked for a new sex partner at in the previous 12 months.**

	Visited	Looked for New Sex Partner
My party	☐	☐
Friends parties	☐	☐
Friend of a friends parties	☐	☐
Parties advertised on the Internet	☐	☐
Parties advertised in a paper or flyer	☐	☐
Other	☐	☐
Looked for no partners at a party last 12 months	☐	☐
Decline	☐	☐

In the "visited" column, mark all the places visited in the past 12 months.

In the "looked for new sex partner" column, mark all places where you looked for someone new in the past 12 months, then press NEXT.

Q104 **Please indicate which telephone chatlines you have visited in the previous 12 months and which you have looked for a new sex partner on in the previous 12 months.**

S8 In the next series of questions we will ask you about [Venue Name] and partners that you may have met here.

Q105 How often do you come to [Venue Name]?

Please answer in only one box.

per week ________

per month ________

per year ________

First time here ________

To decline, type -1 in any one box.

If you are not sure, please estimate or guess.

Q106 For what reason(s) did you come to [Venue Name] today?

- To socialize with friends ☐
- To meet new people ☐
- To "cruise" or meet a new partner ☐
- To drink alcohol ☐
- I'm on a date ☐
- I was hoping to see someone I know ☐
- To dance ☐
- To celebrate ☐
- Other ☐
- Decline ☐

Q107 **Are you alone or with friends here today?**

- Alone ☐
- I came with friends ☐
- My friends and I planned to meet here ☐
- I came alone, and ran into people I knew here ☐
- I came here with my partner ☐
- I planned to meet my partner here ☐
- My friends work here ☐
- Decline ☐

Q108 **Have you ever met someone for the first time here at [Venue Name] that you later had sex with?**

- Yes ☐
- No ☐
- Decline ☐

Q109 **In the past 12 months, how many different people have you met at [Venue Name] that you have had sex with later?**

____

Q110 **Please think last time that you back to the met a partner at [Venue Name].**

How long ago did you meet him or her here? To decline, type -1.

Hours ________________

Days ________________

Weeks ________________

Months ________________

Years ________________

Q111 **Was this person a man or a woman?**

- Man ☐
- Woman ☐
- Decline ☐

Q112 **How old was this person?**

If you don't know, please guess.

Q113 **What was this person's ethnicity?**

- African American/ Black ☐
- Asian/ Pacific Islander ☐
- Caucasian/ White ☐
- Hispanic/ Latino ☐
- Native American/ Native Alaskan/ American Indian ☐
- Other ☐
- Decline ☐

Q114 **What type of partner would you consider this person to have been?**

- unknown-never met before you had sex, won't see again ☐
- onetime-had sex once, could find again if you wanted to ☐
- acquaintance-had sex more than once, but not regularly, do not socialize with ☐
- friend-had sex more than once, but not regularly, but socialize with ☐
- regular- have sex with on a regular basis, but not your main partner ☐
- main- your primary sex partner, "boyfriend", "girlfriend", etc. ☐
- trade- you gave sex for money or other goods or they gave you sex for money or other goods ☐
- decline ☐

Q115 **What types of sexual activity did you have with this person the first time you had sex with him?**

Select as many as apply, then press NEXT.

- gave oral sex using a condom ☐
- gave oral sex without a condom ☐
- received oral sex when wearing a condom ☐
- received oral sex without a condom ☐
- gave anal sex while wearing a condom ☐
- gave anal sex without a condom ☐

received anal sex using a condom ☐

received anal sex without a condom ☐

received fisting ☐

gave fisting ☐

decline ☐

other ☐

Please indicate which other sexual activities you had with this person. ______________________

Q116 How many times in total did you have each of the following sexual activities with this man?

If you don't know, please guess.

Gave oral sex ______________________

Received oral sex ______________________

Top in anal sex with a condom ______________________

Top in anal sex no condom ______________________

Bottom in anal sex with a condom ______________________

Bottom in anal sex no condom ______________________

Q117 What types of sexual activity did you have with this woman the first time you had sex with her?

gave oral sex using a barrier ☐

gave oral sex without a barrier ☐

received oral sex when wearing a condom ☐

received oral sex without a condom ☐

had anal sex while wearing a condom ☐

had anal sex without a condom ☐

had vaginal sex using a condom ☐

had vaginal sex without a condom ☐

decline ☐

other ☐

Q118 **How many times in total did you have each of the following sexual activities with this woman?**

If you don't know, please guess.

Gave oral sex _______

Received oral sex _______

Had vaginal sex with a condom _______

Had vaginal sex no condom _______

Had anal sex with a condom _______

Had anal sex no condom _______

Type a number in each box (even if 0), then press NEXT. To decline type -1.

Q119 **How long after meeting this person did you have sex with him/her?**

Please answer in one time frame only.

Hours _______

Days _______

Weeks _______

Months _______

Years _______

Q120 **Which of the following drugs have used during or just before sex with this person?**

Select all that apply, then press NEXT.

- Alcohol ☐
- Methamphetamine ☐
- Ecstasy ☐
- Poppers ☐
- Ketamine ☐
- GHB ☐
- Cocaine ☐
- Heroin ☐
- Marijuana ☐
- LSD ☐
- Mushrooms ☐

Oxycontin ☐

Vicodin ☐

Valium ☐

Viagra, Cialis, Levitra (not prescribed) ☐

Other drugs ☐

No drugs used ☐

Decline ☐

Q121 Did this person use the same drugs as you during (or just before) sex with you?

Yes ☐

No, but s/he used other drugs ☐

No, s/he did not use drugs ☐

I don't know ☐

Decline ☐

Q122 Do you plan to have a friendship or sexual relationship with this person in the future

Yes ☐

No ☐

Maybe ☐

Decline ☐

Q123 Do you still have sex with this person?

Yes ☐

No ☐

Decline ☐

Q124 Do you still socialize with this person?

Yes ☐

No ☐

Decline ☐

Q125 **Of all the people you have had sex with in the past 12 months, how many do you think have been to [Venue Name] in the past year?**

____

Type the number that you think might have been here in the past 12 months, then press NEXT.

Q126 **If you met someone here at [Venue Name] that you wanted to have sex with today, but you wanted to use a condom during sex, how would you get that condom?**

- I have a condom with me ☐
- A vending machine here ☐
- There are free condoms here ☐
- A nearby store ☐
- A friend who carries condoms ☐
- A nearby vending machine ☐
- I would have sex without a condom ☐
- I would not have sex ☐
- I'm not sure ☐
- Other ☐
- Decline ☐

Q127 **If someone wanted to use recreational drugs at [Venue Name] or near here, where might they go to get them?**

- People sell them here ☐
- Nearby street ☐
- Nearby club ☐
- They would have to bring them from somewhere else ☐
- I don't know ☐
- Other ☐
- Decline ☐

S9 In the next series of questions we will ask about your HIV and sexually transmitted disease history.

Remember that all of your answers are anonymous and you can decline to answer any questions that you do not feel comfortable answering.

Q128 Have you ever had an HIV test?

- Yes ☐
- No ☐
- I'm not sure ☐
- Decline ☐

Q129 How long ago were you last tested for HIV?

Please answer in only one time frame and leave all other boxes blank.

Days ________

Weeks ________

Months ________

Years ________

Q130 What do you believe your current HIV status is?

- Negative ☐
- Positive ☐
- I'm not sure ☐
- Decline ☐

Q131 For what reason(s) do you believe you are HIV negative?

- Recent negative test ☐
- Have only had sex with one other person for 6 months or more ☐
- Have only ever had oral sex ☐
- Have only had oral sex since last HIV test ☐
- Have never had sexual activity without a condom ☐

Have never had sexual activity without a condom since last HIV test ☐

Always the top (insertive partner) during anal sex ☐

Always the top (insertive partner) during anal sex since last HIV test ☐

I believe that I am immune to HIV ☐

I've never had oral, anal, or vaginal sex ☐

Other ☐

Q132 For what reason(s) do you believe you are HIV positive?

Had a positive test result ☐

Physician/ nurse confirmed positive test ☐

Had sex with someone who was HIV positive ☐

I was sick at one point in time and believe it was HIV ☐

Never received a positive test, but think I am infected due to past sexual activity ☐

I have been exposed to an HIV positive persons blood ☐

I was born HIV positive ☐

Other ☐

Decline ☐

Q133 For what reason(s) are you unsure about your HIV status?

Never had an HIV test ☐

Tested negative, but I may have been infected since ☐

Tested positive, but I think the test was wrong ☐

I don't believe that HIV is real ☐

Other ☐

Q134 Have you ever been diagnosed with a sexually transmitted disease by a doctor, nurse or other medical provider?

Yes ☐

No ☐

Decline ☐

Q135 **How many times have you been diagnosed with a sexually transmitted disease?**

____

Q136 **How long ago were you most recently diagnosed with a sexually transmitted disease?**

Please answer in only one time frame and leave all other boxes blank.

Days ______________________

Weeks ______________________

Months ______________________

Years ______________________

Q137 **The last time that you were diagnosed, which sexually transmitted disease did you have?**

- Chancroid ☐
- Chlamydia (The Clam, Gooey Stuff) ☐
- Genital Warts (HPV, Human Papilloma Virus) ☐
- Gonorrhea (Dose, Clap, Drip) ☐
- Hepatitis B ☐
- Herpes ☐
- Lice (pubic lice, crabs) ☐
- Molluscum (Molluscum Contagiosum) ☐
- NGU (Non-gonococcal urethritis) ☐
- Syphilis ☐
- Trichomonas ☐
- Decline ☐
- Other ☐

Q138 **What additional sexually transmitted diseases have you been diagnosed with previously?**

- Chancroid ☐
- Chlamydia (The Clam, Gooey Stuff) ☐
- Genital Warts (HPV, Human Papilloma Virus) ☐
- Gonorrhea (Dose, Clap, Drip) ☐

Hepatitis B ☐
Herpes ☐
Lice (pubic lice, crabs) ☐
Molluscum (Molluscum Contagiosum) ☐
NGU (Non-gonococcal urethritis) ☐
Syphilis ☐
Trichomonas ☐
Other ☐
Decline ☐

S10 In the next series of questions we will ask about your alcohol and drug use over the past 12 months.

We'd like to remind you that all of your answers are anonymous and you can decline to answer any questions that you are too uncomfortable to answer.

Q139 In the past 12 months, which of the following drugs (or alcohol) if any have you have you used?

Alcohol ☐
Methamphetamine ☐
Ecstasy ☐
Poppers ☐
Ketamine ☐
GHB ☐
Cocaine ☐
Heroin ☐
Marijuana ☐
LSD ☐
Mushrooms ☐
Oxycontin ☐
Vicodin ☐
Valium ☐
Viagra, Cialis, Levitra (not prescribed) ☐

Other drugs ☐

No drugs used ☐

Decline ☐

Q140 **In the past 12 months, which drugs (or alcohol) have you used around the time of sexual activity?**

Alcohol ☐

Methamphetamine ☐

Ecstasy ☐

Poppers ☐

Ketamine ☐

GHB ☐

Cocaine ☐

Heroin ☐

Marijuana ☐

LSD ☐

Mushrooms ☐

Oxycontin ☐

Vicodin ☐

Valium ☐

Viagra, Cialis, Levitra (not prescribed) ☐

Other drugs ☐

No drugs used ☐

Decline ☐

Q141 **Which drugs have you used at or just before coming to [Venue Name]?**

Alcohol ☐

Methamphetamine ☐

Ecstasy ☐

Poppers ☐

Ketamine ☐

GHB ☐

Cocaine ☐

Heroin ☐

Marijuana ☐

LSD ☐

Mushrooms ☐

Oxycontin ☐

Vicodin ☐

Valium ☐

Viagra, Cialis, Levitra (not prescribed) ☐

Other drugs ☐

No drugs used ☐

Decline ☐

Q142 **Which drugs have you used with people that you met at [Venue Name] and had sex with?**

Alcohol ☐

Methamphetamine ☐

Ecstasy ☐

Poppers ☐

Ketamine ☐

GHB ☐

Cocaine ☐

Heroin ☐

Marijuana ☐

LSD ☐

Mushrooms ☐

Oxycontin ☐

Vicodin ☐

Valium ☐

Viagra, Cialis, Levitra (not prescribed) ☐

Other drugs ☐

No drugs used ☐

Q143 **Have you ever injected drugs or steroids?**

Select your answer, then press NEXT.

- Yes ☐
- No ☐
- Decline ☐

Q144 **Have you injected drugs or steroids in the past 12 months?**

- Yes ☐
- No ☐
- Decline ☐

Q145 **How long ago was the last time that you injected drugs or steroids?**

Please answer in only one box, leaving the others blank.

Days ________________________________

Weeks ________________________________

Months ________________________________

Years ________________________________

Q146 **In the last 12 months have you shared a syringe with anyone else when injecting drugs or steroids?**

- Yes ☐
- No ☐
- Decline ☐

Q147 **In the last 12 months have you gone to a bar, club or bathhouse in or near Tijuana (TJ)?**

- Bar/ Night Club in TJ ☐
- Bathhouse in TJ ☐
- Have been to none in past 12 months ☐
- Decline ☐

Q148 **What are the names of the bar(s)/night club(s) that you have visited in Tijuana?**

Please type the name(s) in the space below, then press NEXT.

To decline, type "decline".

If you are not sure of the name, please provide any other identifying information (e.g., street name).

____

Q149 **What are the names of the bathhouse(s) you have visited in Tijuana?**

Please type the name(s) in the space below, then press NEXT.

To decline, type "decline".

If you are not sure of the name, please provide any other identifying information (e.g., street name).

____

Q150 **In the past 12 months, have you had sexual contact with someone from Tijuana or a Mexican city or town near Tijuana?**

- Yes ☐
- No ☐
- Decline ☐

Q151 **How many people from the Tijuana area have you had sex with in the past 12 months?**

____

Q152 **Was the person that you had sex with from the Tijuana area a man or a woman?**

- Man ☐
- Woman ☐
- Decline ☐

Q153 **Have you had sex with men, women, or both in the last 12 months from the Tijuana area?**

- Men ☐
- Women ☐
- Both ☐
- Decline ☐

Q154 Have you answered this questionnaire before?

- Yes ☐
- No ☐
- I'm not sure ☐
- Decline ☐

Q155 Where were you when you answered this questionnaire before?

____

Q156 How long ago did you answer this questionnaire?

Please answer in only one box, leaving all others blank.

Days ____

Weeks ____

Months ____

S11 Thank you for helping us with our research.

Your anonymous responses are likely to help researchers at UCSD to better understand how to prevent HIV

transmission within this community.

Q157 If there is any other information you would like to provide to us, please feel free to do so in the space below.

S12 When you reach this screen please hand the PDA back to your interviewer.

S13 Thank you for learning about our study and considering participation!

S14 You have indicated that you no longer wish to participate. Please return the PDA to your interviewer.

S15 Have a wonderful and Safe evening.

INTERVIEWER SCREEN*************************************

I19 Was this your last interview in this venue?

Yes ☐

No ☐

I20 What is the current time entered as HH:MM (24 hour)?

____

I21 Please count the approximate number of patrons in this venue (if under 100) or estimate how full the venue is.

Estimate has not changed since entering venue ☐

Answer by number ☐

Answer by percent ☐

I22 How many men were attending this venue at this time?

____

123 How many women were attending this venue at this time?

____

124 Enter how full the venue was by percent maximum capacity. (Note that you can over 100% if you believe the venue exceeded its maximum capacity). (Based on the names of venues entered we can provide the maximum capacity for the interviewer)

____

I25 What percent of attendees were men?

____

I26 What percent of attendees were women?

____

I27 **Did you observe condom availability in this location?**

Yes ☐

No ☐

I28 **Where did you see condoms available?**

____

I29 **How much did the condom cost (if free $0.00)?**

____

S16 **This is the end of the interview, please select submit to move to a new interview.**
